# Experiences from the Merger of Clinics in the Swedish Public Dental Service - the Employee Perspective

**DOI:** 10.2174/1874210601711010503

**Published:** 2017-09-22

**Authors:** Christina Hassel Gustafsson, Anna-Lena Östberg

**Affiliations:** 1 Public Dental Service, Västra Götaland, Sweden; 2 Department of Behavioral and Community Dentistry, Institute of Odontology, The Sahlgrenska Academy, University of Gothenburg, Göteborg, Sweden; 3 R&D Centre Skaraborg, Skövde, Sweden

**Keywords:** Dental staff, Health facility merger, Job satisfaction, Organization and administration, Questionnaire, Work load

## Abstract

**Objectives:**

The purpose of this study was to investigate the experiences of employees regarding the merger of clinics within the Public Dental Service (PDS), Västra Götaland Region, Sweden.

**Methods:**

Employees (dentists, dental hygienists, dental nurses) affected by both administrative and geographical mergers of dental clinics answered a web-based survey about experiences and effects of the merger process (n = 99, 47%). The Swedish short-form version of “The Nordic Questionnaire for Psychological and Social Factors at Work” (QPSNordic), the QPSNordic-34+ was used. Chi-squared tests and logistic regression analyses were used.

**Results:**

Two thirds of the participants were aged ≥ 50 years. The respondents stated that the reasons for the merger were often made clear (78%). Satisfaction with and involvement in the merger process received lower scores (45%). Work was often perceived as stressful, irrespective of the merger. Job demands and engagement scored positively, but control at work was given a low score (one fifth stated fairly high or high control). Dentists (OR 5.9; 95%, CI 1.1-32.3), but not dental hygienists (OR 2.8; 95%, CI 0.9-9.0), indicated stress significantly more often than dental nurses (reference) (adjusted for age and gender).

**Conclusion:**

Employees in the Public Dental Service (PDS) in a Swedish region had mainly positive experiences after the merger of clinics; however, their involvement in the process was low. Work demands were perceived as high. These findings should be considered when planning mergers in dental organizations.

## 1 INTRODUCTION

Many health care clinics have been merged in recent years, primarily for economic and other efficiency gains [1]. Few mergers have been carried out in dentistry and there is a lack of scientific publications in the field. In recent years, the Public Dental Service (PDS) in the Swedish Västra Götaland Region (VGR) has been reorganized, including the merger of clinics. These have been accomplished either through both financial and physical mergers, or by mere administrative/financial mergers, while keeping the existing premises. Physical mergers have been carried out using one of two models: both clinics have moved to new joint premises or one of the clinics has moved into the other clinic’s existing premises. Some mergers required adaptation of working hours and a form of shift work was introduced. The expected effects were, for example, better utilization of the existing premises, better service to the customer/patient, and reduced fixed costs [1]. The number of clinic managers has, accordingly, decreased, and the clinic managers in merged clinics are now responsible for a larger number of employees.

A number of studies on the reorganization of care have been carried out, revealing implications for the staff. A health care study showed, for example, that the word “reorganization” carried negative associations for nurses [2]. Relocated nurses had a significantly higher level of emotional stress than those who were not relocated [3]. This study found significant positive correlations between the perceived threats, emotional stress and burnout among employees, and a significant negative correlation between feeling threatened and growing professionally. Yet another health care study [4] showed that employee’s resistance to change could be related to uncertainty about which goals the management wanted to achieve, *i.e.*, the reason for the merger. Different perceptions and interpretations of vaguely formulated goals led to greater resistance to change [4] accordingly, the greater the clarity of the management, the smaller the risk of employees making their own interpretations. Good leadership [5], employee participation in the merger process [6], detailed planning prior to the reorganization [7], a professional approach and a common organizational culture [8] have been identified as key factors for success in mergers. No studies within dentistry could be found.

The Swedish Dental Act sets out the requirements for dentistry [9]. It stipulates, among other things, that no patient should have to wait an unreasonably long time to receive care, and that care should be provided on equal terms for all. There has been a shortage of dentists in recent years, especially in rural areas and in smaller towns, and a clearer delegation and distribution of tasks has been developed in many working groups. This has led to further development of the expertise and independent work of both the dental hygienists and the dental nurses. A positive effect of clinic mergers could be better possibilities to make full use of the merged clinics’ combined expertise, thereby facilitating compliance with basic requirements of the Swedish Dental Act, including safety for the patient and care provided at reasonable cost [9]. Scientific studies have shown that the merger of operating units within the health service affects employees and clinic management differently, depending on the reasons stated, how the merger was implemented, and the time from decision to implementation [4-8]. As the trend of mergers of dental clinics continues, there is a need to evaluate how the reorganization of the PDS in the Västra Götaland Region was perceived by the employees and to take advantage of their experiences.

Thus, the purpose of this study was to investigate the experiences of the employees regarding the merger of clinics within the Public Dental Service (PDS) in the Västra Götaland Region. The following specific questions were asked: What were the attitudes of the employees to the merger of the clinics? How did the employees experience the implementation and the effects of the merger of the clinics? What was the psychological and social climate among the employees like?

## 2 MATERIALS AND METHODS

### 2.1 Sample

The sample consisted of employees from PDS clinics in the VGR, where geographic relocation due to clinic mergers took place between 2007 and 2011. An approximate total of 545 employees were affected by administrative mergers, and about 280 of these affected by both administrative and geographical mergers. All these 280 employees were asked to participate in the study and fill out a questionnaire. The Ethics Committee of Göteborg University assessed and approved the protocol (registration no. 023-13) and the PDS management was positive to the study.

### 2.2 Questionnaire

Background questions were asked to describe the participants’ age, gender, profession, and management position, if applicable. The questions about the merger of the clinics covered concerns and hopes before the implementation (*e.g.*, understanding of the merger and feelings about the possibilities to influence and participate), how the mergers were implemented (*e.g.*, the time from information to implementation), and the perceived effects of the mergers. The questions about the merger were formulated on the basis of existing studies in other areas of care [4-7] and had five response options, from the least positive (“do not agree at all”) to the most positive (“agree completely”). The formulations and intelligibility of the merger items were tested on three PDS employees representing the professions in question (dental nurses, dental hygienists, dentists). These persons were not included in the main study.

The Swedish short-form version of “The Nordic Questionnaire for Psychological and Social Factors at Work” (QPSNordic), the QPSNordic-34+, was used [10, 11]. The QPSNordic was developed in collaboration between the Nordic countries and validated in different sectors of working life, such as industrial production, the private service sector, public administration and health care [12]. The QPSNordic-34+ contains 34 questions in the following content areas and conceptual levels: the task level (job demands, control at work, role expectations), the social and organizational level (social interactions, leadership, organizational culture and climate), and the individual level (predictability at work, work motivation, job satisfaction). Each item had five response options, from the least positive (“very seldom/never” or “do not agree at all”), to the most positive (“very often/always” or “agree completely”).

### 2.3 Data Collection

The data were collected during September and October 2013, using a web-based survey [esMakerNX2, 2011] administered by a secretary not involved in the study. After two weeks, the computer program automatically sent a reminder to non-responders. Participation was voluntary and the final database contained no personal identification details.

### 2.4 Data Analysis

The data were analysed using SPSS, the Statistical Package for the Social Sciences, version 22.0 [13]. Descriptive statistics described the sample and questionnaire data. When indicated, Cronbach’s α examined the internal consistency in the QPSNordic-34+ scales. The chi-squared test was used to test associations between professional characteristics and merger details. Bivariate and multivariate logistic regression analyses explored possible associations between the chosen independent variables (professional characteristics and merger details) and one dependent variable: Have you felt stress lately? (one item from the QPSNordic-34+). The dependent variable was dichotomized into two variants: A) response categories 1 (very seldom or never), 2 (fairly seldom) and 3 (sometimes) into “0”, and response categories 4 (fairly often) and 5 (very often or always) into “1” and B) response categories 1 (very seldom or never) and 2 (fairly seldom) into “0”, and response categories 3 (sometimes), 4 (fairly often) and 5 (very often or always) into “1”. When indicated, age, gender and other variables were entered in the regressions as covariates.

## 3 RESULTS

The questionnaire was completed by 99 employees (47%). Table **1** shows the personal and professional characteristics of the participants. More than two thirds of the participants were aged ≥ 50 years and 9 out of 10 were women.

Table **2** gives details of the merger process: one fifth of the respondents had moved and joined another clinic, one fourth had moved into new premises while the majority had remained in their old premises. The number of respondents in clinics with > 20 employees was 28 before but 47 after the mergers and about half had changed their work schedules (50%). The merger process had lasted less than six months for one fourth of the respondents. Almost two thirds had new management in some way (manager and/or coordinator).

Table **3** presents the respondents’ experiences and perceptions of the merger process. The reasons for the merger were mostly made clear, according to the respondents; however, their involvement in and satisfaction with the process were low. The attitude to the merger was more positive as a whole after the completed process (48% before and 61% after the merger being fairly/very positive). Seven out of ten respondents were satisfied with their current schedule and the new routines were considered to work well. However, satisfaction was statistically higher (85%) among those retaining their old schedule, than among those having new working hours (07.00-19.00, 45% satisfied) or “other” (63% satisfied). Employees moving into entirely new premises were significantly (*p* = 0.020) more positive (71% positive) before the merger than those staying in their own old clinic (43%), or those moving to another existing clinic (29%), while no such differences in attitude could be seen after the merger. There were no statistically significant differences related to age, gender or profession with regard to the experience or perceptions of the merger.

Having a managerial position was related to a higher degree of involvement (79% *vs*. 20%, *p* < 0.001) and greater satisfaction with the process (79% *vs*. 39%, *p* = 0.006) and with schedules (93% *vs*. 65%, *p* = 0.037), compared with those not in a managerial position.

The number of respondents reporting stress before and after the merger was similar. However, of those reporting low stress (not at all/little/some) before the merger (n = 76), some were more stressed (fairly or very) after the merger (n = 6) (not in table). Moreover, most of the respondents who were highly stressed before the merger (n = 24) reported lower stress after the merger (n = 15) (not in tables).

The results from the QPSNordic-34+ are presented in Tables **4**-**6**, with the proportions in the extreme categories combined (response 1 combined with response 2, and response 4 combined with response 5). Table **4** presents items on *the task level.* Job demands yielded positive scores that is, the respondents had great perceptions of own ability and positive challenges. The scores and mean values for work load indicate that the respondents sometimes or often had too much to do. The scores were positive for role expectations, as a whole. The items on control at work received lower scores and only around one fifth reported fairly high or high control.

Table **5** includes items on the social and organizational level in the QPSNordic-34+. The items about social interactions received positive scores overall, especially with regard to expected support from colleagues (mean value 4.06) and friends/family (mean value 4.01). One third often perceived having positive leadership. The working climate was perceived to be good and inequalities in the treatment of staff were rarely reported. Rewards for a job well done received the least positive answers.

Items on *the individual level* in the QPSNordic-34+ are shown in Table **6**. One fifth of the respondents reported rumors of further changes in the workplace. Good job satisfaction was indicated with perceived high skill levels (76%) and commitment to work (72% often enjoying immersing themselves in their work). About one fourth (24%) stated fairly much or very much stress lately.

Two QPSNordic-34+ subscales contained > 2 items and were considered possible to test for internal consistency, namely “control at work” (4 items, Cronbach’s α 0.75) and “support at work” (3 items, Cronbach’s α 0.76).

In bivariate and multivariate logistic regressions, using “Did you feel stressed lately?”, dichotomized into little/some *vs.* high levels of stress as the outcome, there were no statistically significant differences according to age, profession, moving category, managerial position, number of employees or working hours. When dichotomizing “Did you feel stressed lately?” into little *vs*. some/high levels of stress as the outcome, dentists (OR 6.2, 95% CI 1.6-23.9) and dental hygienists (OR 3.3, 95% CI 1.1-9.8) indicated more stress than dental nurses (reference category). When adjusting for age and gender, statistical significance remained for dentists (OR 5.9, 95% CI 1.1-32.3) but not for dental hygienists (OR 2.8, 95% CI 0.9-9.0).

## 4 DISCUSSION

The most important findings in this study were that employees in the Public Dental Service (PDS) in the Swedish Västra Götaland Region mostly experienced that new routines worked well after the merger of clinics however their satisfaction with and involvement in the merger process scored less positively. Employees in managerial positions had more positive experiences of the merger process.

The respondents perceived their job demands as positive; however, control at work was less positive. Dentists had the highest risk of perceived stress.

The response rate in the study was in line with similar web-based surveys [14]. The reason for not responding could be “survey fatigue”, as numerous surveys are carried out nowadays [15]. Also, some employees may not have felt sure that their employer would not get access to their answers. Another explanation could be that the survey was distributed electronically and aimed at reaching all employees; however, some employees may not have checked their e-mails regularly. The sample was fairly small which resulted in small numbers in some subgroups; hence, true statistically significant differences may not have been found (Type II error). For those participating, the risk of memory bias and social desirability should be considered, as in all self-reported measures [16]. The representativeness of the total sample is difficult to judge, as the characteristics of the non-responders are unknown. People in managerial positions were more likely to answer-15% of the participants indicated this, while only 9.6% in the PDS as a whole holds such a position (communication with the PDS administration). Due to their involvement in the merger process they may have been more interested in the subject.

The validated instrument QPSNordic-34+ was used to enable comparisons with similar studies and also to enable follow-up. The short form was used, as the original QPSNordic comprises 123 items and was considered too comprehensive and could possibly have lowered the participation rate and/or increased the number of internal dropouts. A drawback was that most of the subscales in the instrument could not be tested, except for the control (4 items) and support dimensions (3 items); however, both showed acceptable internal consistency [17] and corresponded to the reliability tests in the development of the instrument [12]. Another aspect was that the response options in the QPSNordic-34+ comprised a middle alternative often criticized as being a “convenience choice” [17, p. 71]. Still, many individuals experience difficulties when having to choose between extremes, which may lead to high internal dropout rates. We do not know whether this was the case in the current study, but internal dropouts were low overall.

The age distribution among those participating mirrored the situation in the PDS in the region as a whole (communication with the PDS administration). Many employees may have worked in the organization for a long time with commitment to their work, while younger employees may not have the same perspective. The proportion of women was high (93%), which also corresponds to the gender distribution in the PDS as a whole (general dental care 90%, specialist dental care 85%) (communication with the PDS administration).

The working conditions had changed considerably for most respondents, such as new working hours and new management. However, the reasons for the merger had been well communicated according to the majority of respondents, which Roald and Edgren found to be important to minimize resistance to change [4]. Still, participation in the process could have been better for goal fulfillment in the merger process [6]. Individuals in managerial positions indicated more positive experiences of the merger process, which may be considered natural, as they were involved throughout the implementation of the mergers. Similar findings were found in a US study [18]. It is important that managers at all levels communicate information and involve employees as much as possible in the planning and decisions about changes to the organization [19].

As a whole, the frequency distributions and mean values on specific items of the QPSNordic-34+ were similar to the figures from the method development studies [12] and the validation study by Wännström *et al.* [20]. However, the items on control at work received lower scores than in the reference material and also compared with results from a study on health care [11], especially for “Can you determine your pace of work?”, where 21% in the current study reported “very often or fairly often” or “to a high degree”, compared with 41% in the reference material [12], and for the item, “Can you decide when to take a break?” (23% vs. 44%). Taking a break is of course regulated by the patient work in dentistry, but the work schedule is, nevertheless, strict according to our results, and should perhaps be subjected to greater employee control. The economic results are carefully followed in dental clinics and might influence the employees’ experiences. It is noteworthy that combinations of high work demands and low decision latitude could be potential risk factors for mental distress, according to a meta-analytic review [21].

A key finding was that stress levels were rather high among the employees, both before and after the merger. Dentists and dental hygienists reported high stress more often than dental nurses; however, adjustments for age and gender modified the outcome for dental hygienists. A review found multiple demands on dentists [22]. These requirements must often be met under time pressure. An interesting finding was that not exactly the same people reported stress before and after the merger. Individuals may have different reasons for perceiving stress and changes could be stressful *per se* [22]. Another notable finding was the great commitment among the respondents, with many deeply engaged in their work and more than two thirds enjoying immersing themselves in their work. This is in line with earlier findings with high levels of work engagement in both dentists and dental hygienists [23, 24]. The corresponding figure in the method development study of QPSNordic, representing a variety of different sectors of working life, was only one third of the respondents [12]. A high level of work engagement in relation to job demands might entail a higher risk for burnout, meaning feelings of emotional and physical exhaustion coupled with a sense of frustration and failure [25]. This was found in dental staff [26] and in a recent review the cause was recognized as multifactorial, including job strain and long working hours [27]. Therefore, these aspects should be well considered when developing dental work conditions.

## CONCLUSION

In conclusion, employees in the Public Dental Service (PDS) in a Swedish region had mostly positive experiences after the merger of clinics; however, their satisfaction with and involvement in the merger process was less positive. Work was often perceived as stressful, regardless of the merger. Job demands and engagement received positive scores, but control at work was experienced as less positive. These findings should be considered when planning mergers in dental organizations.

## Figures and Tables

**Table 1:** Employees’ personal and professional characteristics.

		**n**	**%**
Age	-40 years	10	10
	40-49 years	19	19
	50-59 years	40	41
	60- years	29	30
Gender	Woman	90	93
Profession	Dentist	16	17
	Dental hygienist	21	22
	Dental nurse/else*	59	61
Managing position	Yes	15	16

*including a few medical secretaries, receptionists and orthodontic assistants (n=5) Missing data 0-2%

**Table 2:** Details from the mergers of PDS clinics.

		n	%
Moving category	Moved to another existing clinic’s premises	18	19
	Moved to new premises	24	26
	Remained in old clinic’s premises	51	55
Employees in old clinic	< 10 people	28	30
	10-20 people	39	40
	> 10 people	28	30
Employees in new clinic	< 10 people	8	8
	10-20 people	40	42
	> 10 people	47	50
Working hours after merger (personal)	Same as before	48	50
	7 am to 19 pm	30	31
	Other	18	19
Time from decision to merger	< 6 months	23	26
	6-12 months	32	36
	> 12 months	34	38
Clinic managing after merger	Same clinic manager and same coordinator as before	38	40
	New clinic manager, same coordinator	24	25
	Same clinic manager, new coordinator	7	7
	Both new clinic manager and new coordinator	27	28

Missing data 0-6%

**Table 3:** The employees’ perceptions of the implementation of the merger. Proportions of the participants in percentages (%).

	Not at all/ Very or Fairly Little	Some	Fairly or Very Much
Was the reason for the merger of the clinics made clear?	8	14	78
How well did you know your future colleagues before the merger?	17	25	58
To what extent do you feel that you were involved in the process of the merger?	45	25	30
Are you satisfied with the way the merger was carried out?	28	27	45
Are you satisfied with your current schedule?	11	20	69
Did you feel stressed before the merger?	47	29	24
Did you feel stressed after the merger?	55	22	23
My attitude to the merger before the implementation was positive	19	33	48
My attitude to the merger after the implementation is positive	11	28	61
The development and adaptation of new common routines have worked well	9	21	70

The mean values are based on the 5-point scale. Missing data 1-6%

**Table 4:** Frequencies, means and standard deviations of items at *the task level* in QPSNOrdic-34+.

**Content areas**	**Item**	**Very or Fairly Seldom/Little (%)**	**Sometimes/Some (%)**	**Very or Fairly Often/Much (%)**	**Mean (SD)**
Job demands	Is your workload unevenly distributed so that the work is piling up?	30	48	22	2.87 (0.85)
	Do you have too much to do?	14	54	32	3.23 (0.78)
	Is your job too difficult for you?	78	20	2	1.95 (0.76)
	Do you perform tasks for which you would need more training?	61	38	1	2.06 (2.44)
	Are your knowledge and skills useful in your work?	0	4	96	4.43 (0.58)
	Does your work imply positive challenges?	9	34	57	3.65 (0.94)
Role expectations	Are there clearly defined goals for your work?	6	20	74	3.91 (0.90)
	Do you know exactly what is required of you at work?	0	9	91	4.32 (0.64)
	Are there inconsistent demands on you from two or more people?	54	36	10	2.37 (0.95)
Control at work	Can you influence your amount of work?	39	42	19	2.65 (1.02)
	Can you yourself determine your pace of work?	45	34	21	2.60 (1.08)
	Can you decide when to take a break?	63	14	23	2.27 (1.28)
	Can you influence decisions that are important for your work?	35	42	23	2.83 (1.05)

Missing data 1-4%

**Table 5:** Frequencies, means and standard deviations of items at *the organizational level* in QPSNOrdic-34+.

**Content areas**	**Item**	**Very or Fairly Seldom/Little (%)**	**Sometimes/Some (%)**	**Very or Fairly Often/Much (%)**	**Mean (SD)**
Social interactions	If you need, do you get support and help with your work from your colleagues?	4	17	79	4.06 (0.88)
	If you need, do you get support and help with your work from your nearest manager?	13	23	64	3.77 (1.11)
	Do you get appreciation for your work performance from your nearest manager?	27	33	40	3.17 (1.22)
	Do you feel that you can get support from your friends / your family when there are problems at work?	10	17	73	4.01 (0.99)
	Do you appreciate to be part of your team?	8	17	75	3.96 (0.99)
	Is your team good at solving problems?	9	26	65	3.70 (0.91)
	Are the employees in your workplace encouraged to make improvements?	13	34	53	3.51 (1.00)
	Is there sufficient communication in your workplace?	15	33	52	3.45 (1.03)
	Have you noticed disturbing conflicts between colleagues?	38	45	17	2.73 (0.91)
Leadership	Does your manager encourage you to participate in important decisions?	23	34	33	2.96 (1.10)
	Does your manager help you to develop your skills?	29	38	33	3.08 (1.11)
Organizational culture and climate	Is the climate in your workplace encouraging and supportive?	17	26	57	3.51 (1.02)
	Is the climate in your workplace relaxed and pleasant?	24	22	54	3.40 (1.10)
	Is the climate in your workplace rigid and rule-governed?	40	36	24	2.84 (1.09)
	Have you noticed any inequalities in the treatment of women and men in your workplace?	86	10	4	1.58 (0.83)
	Have you noticed any inequalities in the treatment of older and younger workers in your workplace?	84	14	2	1.60 (0.84)
	Are employees rewarded for a job well done in your workplace (money, encouragement)?	54	33	13	2.33 (1.09)
	To what extent is the management interested in the health and wellbeing of the staff?	16	35	49	3.45 (1.11)

Missing data 2-5%

**Table 6:** Frequencies, means and standard deviations of items at *the individual level* in QPSNOrdic-34+.

**Content areas**	**Item**	**Very or Fairly Seldom/Little (%)**	**Sometimes/Some (%)**	**Very or Fairly Often/Much** **%**	**Mean (SD)**
Predictability at work	Do you know a month in advance what kind of tasks you will have?	17	14	69	3.73 (1.20)
	Are there rumors of changes in the workplace?	47	33	20	2.60 (0.98)
Perceived mastery/skill	Are you satisfied with your ability to solve problems at work?	3	21	76	3.90 (0.70)
Interaction between work and private life	I enjoy to go completely up in my work most of the time	10	18	72	3.83 (0.99)
	The greatest satisfaction of my life comes from my work	41	44	16	2.60 (0.92)
	Did you feel stressed lately?	45	31	24	2.68 (1.25)

Missing data 2-5%
